# Changes in the Expression of Aquaporin-3 in the Gastrointestinal Tract Affect Drug Absorption

**DOI:** 10.3390/ijms20071559

**Published:** 2019-03-28

**Authors:** Nobutomo Ikarashi, Chika Nagoya, Risako Kon, Satoshi Kitaoka, Sayuri Kajiwara, Masayo Saito, Akane Kawabata, Wataru Ochiai, Kiyoshi Sugiyama

**Affiliations:** 1 Department of Biomolecular Pharmacology, Hoshi University, 2-4-41 Ebara, Shinagawa-ku, Tokyo 142-8501, Japan; 2 Department of Clinical Pharmacokinetics, Hoshi University, 2-4-41 Ebara, Shinagawa-ku, Tokyo 142-8501, Japan; c.nagoya1226@gmail.com (C.N.); s-kitaoka@hoshi.ac.jp (S.K.); 89wwrol314@ezweb.ne.jp (S.K.); saitom-pha@h.u-tokyo.ac.jp (M.S.); akn910417@gmail.com (A.K.); w-ochiai@hoshi.ac.jp (W.O.); 3 Department of Functional Molecular Kinetics, Hoshi University, 2-4-41 Ebara, Shinagawa-ku, Tokyo 142-8501, Japan; sugiyama@hoshi.ac.jp

**Keywords:** aquaporin, Caco-2 cell, membrane fluidity, drug absorption

## Abstract

Aquaporin-3 (AQP3) plays an important role in water transport in the gastrointestinal (GI) tract. In this study, we conducted a Caco-2 cell permeability assay to examine how changes in the expression and function of AQP3 affect the rate at which a drug is absorbed via passive transport in the GI tract. When the function of AQP3 was inhibited by mercuric chloride or phloretin, there was no change in warfarin permeability. In contrast, when the expression of AQP3 protein was decreased by prostaglandin E_2_ (PGE_2_) treatment, warfarin permeability increased to approximately twice the control level, and membrane fluidity increased by 15%. In addition, warfarin permeability increased to an extent comparable to that after PGE_2_ treatment when cell membrane fluidity was increased by 10% via boric acid/EDTA treatment. These findings suggest the possibility that the increased drug absorption under decreased AQP3 expression was attributable to increased membrane fluidity. The results of this study demonstrate that the rate of water transport has little effect on drug absorption. However, our findings also indicate that although AQP3 and other similar transmembrane proteins do not themselves transport drugs, changes in their expression levels can cause changes in cell membrane fluidity, thus affecting drug absorption rates.

## 1. Introduction

Aquaporins (AQPs) play an important role in water transport in the body [1]. AQPs are membrane proteins with six transmembrane domains and a molecular weight of approximately 30 kDa [2]. These proteins have two asparagine–proline–alanine (Asn-Pro-Ala; NPA) boxes and allow water and glycerol molecules to selectively pass through the membrane along an osmotic gradient [3,4,5]. There are currently 13 known AQPs, AQP0 through AQP12, which are expressed in various organs [6]. Many members of the AQP family are expressed in the gastrointestinal (GI) tract [7,8,9,10,11,12]. Among these, AQP3 is known to be the most abundantly expressed in the mucosal epithelial cells of the GI tract. It has become clear that diarrhea and constipation can be caused by changes in the expression or function of AQP3, indicating that AQP3 is a very important functional protein for water transport in the GI tract [13,14,15,16,17,18].

The GI tract is a site not only of water absorption but also of drug absorption. The rate of drug absorption in the GI tract is greatly influenced by the expression and function of the excretory transporter P-glycoprotein (P-gp) as well as the drug-metabolizing enzyme cytochrome P450 (CYP) [19]. For example, when the expression of P-gp is increased by rifampicin, GI absorption of P-gp substrates, such as quinidine and digoxin, decreases [20,21,22]. Furthermore, when the expression of CYP in the GI tract is increased by St. John’s wort, GI absorption of CYP substrates, such as cyclosporin and tacrolimus, decreases [23,24]. Accordingly, drug transporters and drug-metabolizing enzymes present in the GI tract have emerged as active research topics, and their importance with regards to drug absorption has received substantial attention. However, there have been no discussions regarding how drug absorption rates change in response to changes in water transport rates resulting from altered function and expression of AQPs in the GI tract. The present study focused on AQP3, which is predominantly expressed in the GI tract, and investigated how drug absorption rates change when the function and expression of AQP3 are altered. In particular, the human intestinal epithelial cell line Caco-2, which is frequently used in experiments on GI absorption of drugs [25], was used to analyze the effects of changes in the function and expression of AQP3 on drug absorption rates. In addition, an investigation regarding the underlying mechanism by which decreases in AQP3 expression change drug permeability was undertaken.

## 2. Results

### 2.1. Changes in the Expression and Function of AQP3 during Caco-2 Cell Differentiation

When Caco-2 cells are plated in Transwell plates, transepithelial electrical resistance (TEER) increases with the formation of tight junctions. Therefore, we monitored TEER to confirm the formation of tight junctions during Caco-2 cell differentiation while examining changes in the expression and function of AQP3.

**Figure 1:**
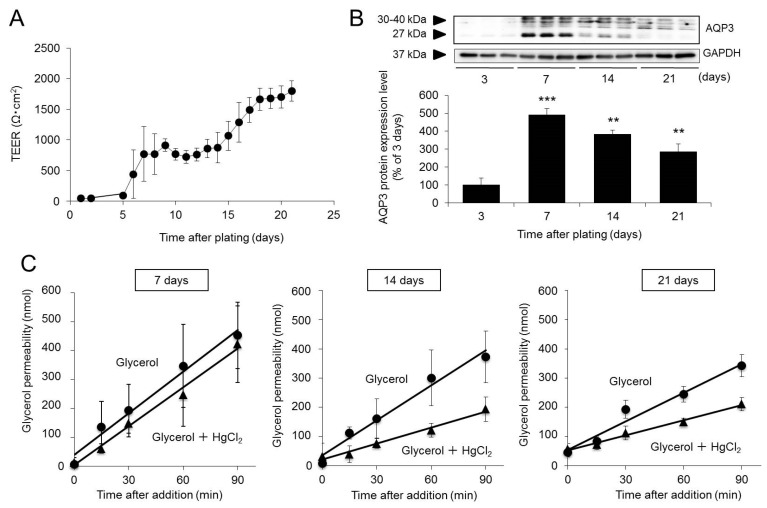
Changes in the expression and function of Aquaporin-3 (AQP3) during Caco-2 cell differentiation. Caco-2 cells were plated in Transwell plates and incubated for 3, 7, 14, or 21 days. (**A**) The integrity of the monolayer was measured by determining the TEER. (**B**) The protein expression of AQP3 was analyzed by western blotting and normalized to glyceraldehyde-3–phosphate dehydrogenase (GAPDH) expression. The mean levels of AQP3 protein expression on day 3 of incubation are indicated as 100%. (**C**) Glycerol alone or a combination of glycerol and HgCl_2_ was added to the apical side of the Transwell plate. The concentration of glycerol on the basal side was measured to calculate the permeated amount of glycerol. The data are presented as the mean ± SD for six experiments. Dunnett’s test: ** *p* < 0.01 and *** *p* < 0.001 vs. the day 3 value.

TEER remained very low until day 5 of incubation and then rapidly increased from day 6 onwards, reaching 800 Ω·cm^2^ on day 7. TEER again increased from day 15 onwards, reaching approximately 1800 Ω·cm^2^ on day 21. These results are consistent with previous reports [26] and confirm that in this experiment, Caco-2 cells differentiated into small intestinal epithelial-like cells by day 21 of incubation and formed a membrane consisting of a monolayer of cells with tight junctions (Figure 1A).

The protein expression of AQP3 in Caco-2 cells peaked on day 7 of incubation and then declined with time. However, the expression of AQP3 on day 21 was significantly elevated, approximately threefold higher than that on day 3 (Figure 1B).

Since glycerol is transported by AQP3, the function of AQP3 can be analyzed by measuring the amount of glycerol that permeates through the membrane [27,28]. In addition, glycerol transport can be inhibited by HgCl_2_ [29]. In this study, we used glycerol and HgCl_2_ to examine the function of AQP3 during the differentiation of Caco-2 cells. When glycerol was added alone, the apparent permeability coefficient (P_app_) of glycerol peaked on day 7 and then declined, consistent with changes in AQP3 expression. In contrast, when HgCl_2_ was added, the P_app_ of glycerol remained unchanged on day 7 of incubation but then decreased by 50–60% on days 14 and 21 due to HgCl_2_ treatment (Figure 1C and Table 1).

The above findings indicate that Caco-2 cells incubated for 21 days exhibit well-formed tight junctions with abundant AQP3 expression and are thus useful as experimental models for studying the effect of AQP3 inhibition on membrane permeability to drugs.

### 2.2. Effect of Inhibition of AQP3 Function on Warfarin Permeability

Warfarin is absorbed by passive transport in the GI tract and does not serve as a substrate for either P-gp or CYP [30]. We used warfarin as a target drug and examined the effect of inhibition of AQP3 function on GI tract drug permeability.

**Figure 2:**
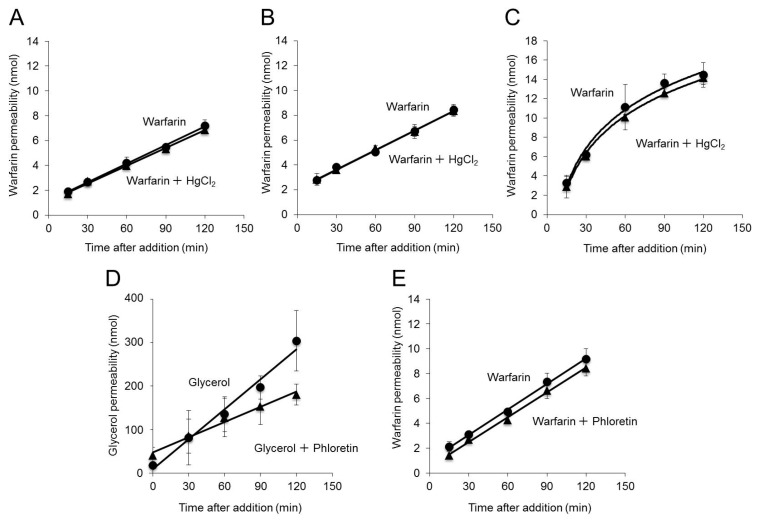
Effect of inhibition of AQP3 function on warfarin permeability. Caco-2 cells were plated in Transwell plates and incubated for 21 days. (**A**) Warfarin alone or a combination of warfarin and HgCl_2_ was added to the apical side of the Transwell plate. (**B**) Warfarin alone or a combination of warfarin and HgCl_2_ was added to the apical side of the Transwell plate, and mannitol was added to the basal side. (**C**) Warfarin alone or a combination of warfarin and HgCl_2_ was added to the apical side, and the Transwell plate was shaken on a plate mixer. (**D**) Glycerol alone or a combination of glycerol and phloretin was added to the apical side of the Transwell plate. (**E**) Warfarin alone or a combination of warfarin and phloretin was added to the apical side of the Transwell plate. The concentration of warfarin or glycerol on the basal side was measured. The data are presented as the mean ± SD for six experiments.

The P_app_ of warfarin for cells treated with warfarin and HgCl_2_ was equal to that for cells treated with warfarin alone (Figure 2A and Table 2). Inhibition of AQP3 function did not affect permeability, even when mannitol was added to the basal side to create an osmotic gradient (Figure 2B and Table 2). Even when the plate was shaken to reduce the stagnancy of the water layer, the P_app_ of warfarin remained unchanged (Figure 2C and Table 2). Furthermore, there was no effect on warfarin permeability even in the experimental system in which phloretin was added to inhibit AQP3 function by approximately 50% (Figure 2D,E).

These findings indicate that inhibition of AQP3 function does not affect warfarin permeability.

### 2.3. Effect of Inhibition of AQP3 Function on Permeability to Various Drugs

Since warfarin is lipophilic and highly permeable, it is considered a Class II drug according to the Biopharmaceutics Classification System (BCS) [31]. We next examined the effects of inhibition of AQP3 function on permeability to drugs with properties different from those of warfarin. In this study, we examined antipyrine (Class I), atenolol (Class II), and furosemide (Class III) permeability when AQP3 function was inhibited by 60% via HgCl_2_ treatment.

**Figure 3:**
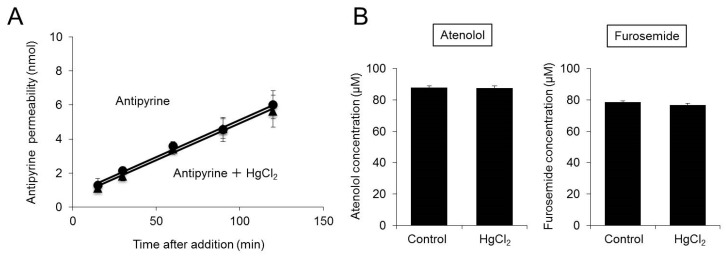
Effect of inhibition of AQP3 function on permeability to various drugs. Caco-2 cells were plated in Transwell plates and incubated for 21 days. (**A**) Antipyrine alone or a combination of antipyrine and HgCl_2_ was added to the apical side of the Transwell plate. The concentration of antipyrine on the basal side was measured. (**B**) Atenolol alone or a combination of atenolol and HgCl_2_ was added to the apical side of the Transwell plate. Furosemide alone or a combination of furosemide and HgCl_2_ was added to the apical side of the Transwell plate. At 120 min after addition, the concentration of atenolol or furosemide on the apical side was measured. The data are presented as the mean ± SD for six experiments.

The P_app_ of antipyrine after the addition of antipyrine and HgCl_2_ was 21.5 ± 2.9 × 10^−6^ cm/s, approximately equal to that after the addition of antipyrine alone (21.4 ± 1.9 × 10^−6^ cm/s) (Figure 3A).

The concentration of residual atenolol on the apical side 120 min after the addition of atenolol and HgCl_2_ was approximately equal to that after the addition of atenolol alone (Figure 3B).

The concentration of residual furosemide on the apical side 120 min after the addition of furosemide and HgCl_2_ was approximately equal to that after the addition of furosemide alone (Figure 3B).

These findings indicate that inhibition of AQP3 function by approximately 60% does not affect permeability to drugs.

### 2.4. Effect of Decreased AQP3 Expression on Permeability to Warfarin/Antipyrine

Prostaglandin E_2_ (PGE_2_) causes a marked, rapid decrease in the protein expression of AQP3 in the HT-29 cell line, which is derived from human colon cancer [16,18]. We therefore used PGE_2_ to decrease AQP3 expression and examined the effect of decreased AQP3 expression on drug permeability.

**Figure 4:**
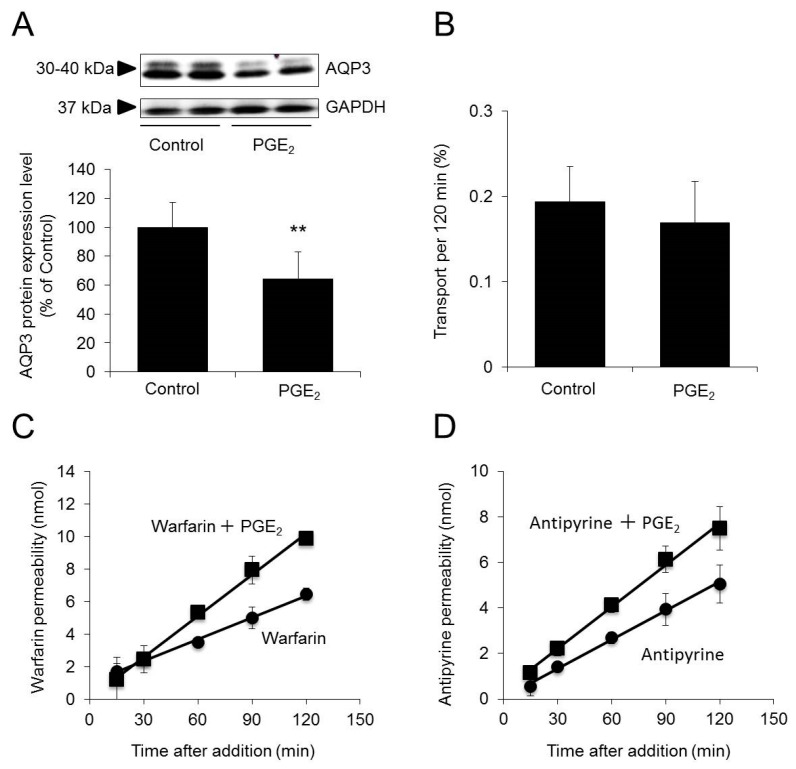
Effect of decreased AQP3 expression on permeability to warfarin/antipyrine. Caco-2 cells were plated in Transwell plates and incubated for 21 days. (**A**) Prostaglandin E_2_ (PGE_2_) was added to the apical side of the Transwell plate. At 1 h after treatment, the protein expression of AQP3 was analyzed by Western blotting and normalized to glyceraldehyde-3–phosphate dehydrogenase (GAPDH) expression. The mean levels of AQP3 protein expression in the control group were set as 100%. (**B**) Lucifer yellow alone or a combination of PGE_2_ and lucifer yellow was added to the apical side of the Transwell plate. At 2 h after treatment, the concentration of lucifer yellow on the basal side was measured. (**C**) Warfarin alone or a combination of warfarin and PGE_2_ was added to the apical side of the Transwell plate. The concentration of warfarin on the basal side was measured. (**D**) Antipyrine alone or a combination of antipyrine and PGE_2_ was added to the apical side of the Transwell plate. The concentration of antipyrine on the basal side was measured. The data are presented as the mean ± SD for six experiments. Student’s *t*-test: ** *p* < 0.01 vs. the control value.

At one hour after the addition of PGE_2_, the protein expression levels of AQP3 in Caco-2 cells were 30% lower than the control levels (Figure 4A).

When warfarin was added in combination with PGE_2_, the P_app_ of warfarin was increased by approximately twofold, to 42.7 ± 5.3 × 10^−6^ cm/s, compared with that when warfarin alone was added (25.2 ± 1.7 × 10^−6^ cm/s) (Figure 4C). When antipyrine was added in combination with PGE_2_, the P_app_ of antipyrine was increased by approximately 1.5-fold, to 30.9 ± 3.2 × 10^−6^ cm/s, compared with that when antipyrine alone was added (21.4 ± 1.8 × 10^−6^ cm/s) (Figure 4D).

We also examined the effect of decreased AQP3 expression on tight junctions using lucifer yellow [25] and found no changes in tight junctions even after the addition of PGE_2_ (Figure 4B).

These results indicate that permeability to drugs increases when AQP3 expression is decreased.

### 2.5. Effect of Decreased P-gp Expression on Permeability to Warfarin/Antipyrine

The results described thus far demonstrate that drug permeability remains unchanged when the function of AQP3 is inhibited but increases when the expression of AQP3 is decreased. This indicates that water permeability does not affect drug permeability. We further noted that permeability to warfarin and antipyrine increase when the expression of AQP3 was decreased. We hypothesized that changes in the expression of membrane proteins, including AQP3, resulted in changes in membrane fluidity, thus altering drug permeability. To test this hypothesis, we examined whether drug permeability changed when the expression of P-gp, a membrane protein, was decreased. Specifically, we examined whether permeability increased for warfarin and antipyrine, which are not P-gp substrates, when P-gp expression was decreased by indomethacin treatment [32].

**Figure 5:**
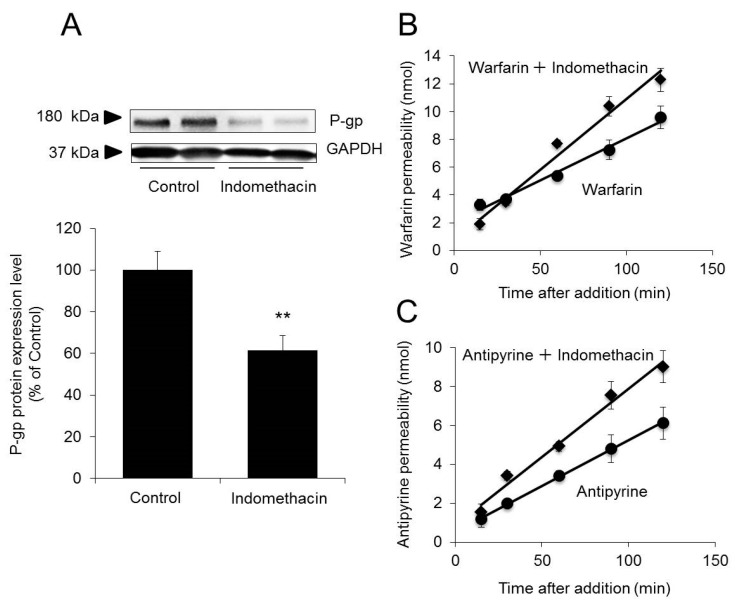
Effect of decreased P-glycoprotein (P-gp) expression on permeability to warfarin/antipyrine. Caco-2 cells were plated in Transwell plates and incubated for 21 days. (**A**) Indomethacin was added to the apical side of the Transwell plate. After 2 days, the protein expression of P-gp was analyzed by western blotting and normalized to GAPDH expression. The mean levels of P-gp protein expression in the control group were set as 100%. (**B**) Warfarin alone or a combination of warfarin and indomethacin was added to the apical side of the Transwell plate. The concentration of warfarin on the basal side was measured. (**C**) Antipyrine alone or a combination of antipyrine and indomethacin was added to the apical side of the Transwell plate. The concentration of antipyrine on the basal side was measured. The data are presented as the mean ± SD for six experiments. Student’s *t*-test: ** *p* < 0.01 vs. the control value.

When indomethacin was added to Caco-2 cells, P-gp expression levels decreased by 40% compared with the control levels (Figure 5A). The P_app_ of warfarin in cells pretreated with indomethacin before warfarin treatment was 51.5 ± 4.9 × 10^−6^ cm/s, approximately 1.8-fold higher than that in cells treated with warfarin alone (28.8 ± 4.4 × 10^−6^ cm/s) (Figure 5B). Similarly, the P_app_ of antipyrine in cells pretreated with indomethacin before antipyrine treatment was 35.4 ± 2.3 × 10^−6^ cm/s approximately 1.7-fold higher than that in cells treated with antipyrine alone (21.4 ± 1.8 × 10^−6^ cm/s) (Figure 5C).

The above findings indicate that permeability to warfarin and antipyrine increases when P-gp expression is decreased, as observed when AQP3 expression is decreased.

### 2.6. Effects of Increased Membrane Fluidity on Warfarin Permeability

It has been reported that decreases in P-gp expression alter the conformation of the membrane, resulting in increased membrane fluidity [33]. It is also known that increases in membrane fluidity also increase membrane drug permeability [34]. Therefore, we examined whether the increased drug permeability due to decreased AQP3 expression was attributable to increased membrane fluidity.

**Figure 6:**
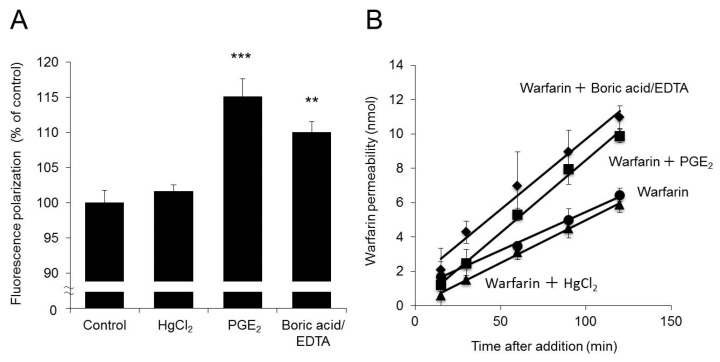
Effects of increased membrane fluidity on warfarin permeability. Caco-2 cells were plated in Transwell plates and incubated for 21 days. (**A**) HgCl_2_, PGE_2_, or boric acid/EDTA was added to the apical side of the Transwell plate. The fluorescence polarization of 1,6-diphenyl-1,2,5-hexatriene (DPH) was then calculated and expressed as a percentage of the control value, which was set as 100%. (**B**) Warfarin alone or a combination of warfarin and HgCl_2_, warfarin and PGE_2_, or warfarin and boric acid/EDTA was added to the apical side of the Transwell plate. The concentration of warfarin on the basal side was measured. The data are presented as the mean ± SD for six experiments. Dunnett’s test: ** *p* < 0.01 and *** *p* < 0.001 vs. the control value.

When AQP3 expression was decreased by 30% by the addition of PGE_2_, membrane fluidity increased by 15% compared with the control level. In addition, when boric acid/EDTA, an agent known to increase membrane fluidity [34], was added, membrane fluidity increased by 10% (Figure 6A).

The P_app_ of warfarin in cells treated with boric acid/EDTA and warfarin was 64.7 ± 6.9 × 10^−6^ cm/s, approximately twofold higher than that in cells treated with warfarin alone (33.3 ± 5.1 × 10^−6^ cm/s). The extent of this increase was comparable to that caused by PGE_2_ treatment (Figure 6B).

The above findings indicate the possibility that decreases in AQP3 expression due to PGE_2_ treatment increase membrane fluidity, resulting in increased membrane permeability to warfarin.

## 3. Discussion

When incubated in a Transwell plate for 21 days, Caco-2 cells differentiate into small intestinal epithelial-like cells. As observed in the small intestine, differentiated Caco-2 cells express transporters such as P-gp and organic anion transporting polypeptide (OATP); thus, these cells are widely used for assessments of drug absorption in the small intestine [25]. Caco-2 cells express AQPs and are frequently used in relevant studies, especially those on AQP3 [35,36,37]. In this study, we used Caco-2 cells to investigate the role of AQP3 in the GI absorption of drugs.

We examined the expression and function of AQP3 during Caco-2 cell differentiation and found that the protein expression of AQP3 in Caco-2 cells peaked on day 7 of incubation and then declined with time; however, even on day 21, expression remained approximately threefold higher than that observed on day 3 (Figure 1A). It has been reported that AQPs not only mediate the permeation of water and glycerol but also play an important role in cell growth [38]. From this perspective, our findings indicated that AQP3 expression levels peaked when the cells started to differentiate on day 7, after which a certain level was maintained until the cells had completely differentiated by day 21.

We examined whether AQP3 expressed in Caco-2 cells functioned normally using HgCl_2_. HgCl_2_ inhibits the AQP3-mediated permeation of water and glycerol by binding to Cys-11, which is located near the NPA box [29]. In this study, we assessed the function of AQP3 during the differentiation process by examining glycerol permeability. Evaluation of P_app_ showed that upon addition of glycerol alone, the permeability decreased with time from day 7 to day 21 after plating (Figure 1C and Table 2). This effect appeared to be correlated with changes in the protein expression levels of AQP3 (Figure 1B). HgCl_2_ treatment did not reduce the P_app_ of glycerol on day 7 of incubation; however, on days 14 and 21 of incubation, HgCl_2_ inhibited glycerol permeability, with similar rates of inhibition on both days (Figure 1C and Table 2). These findings suggest the possibility that tight junctions were not sufficiently well-formed by day 7 of incubation (Figure 1A), enabling glycerol to pass through intercellular spaces in addition to being transported by AQP3. However, the observations on days 14 and 21 of incubation suggest that by this time, tight junctions were sufficiently formed for glycerol to be transported by AQP3 without passing between cells (Figure 1A).

We also examined how drug permeability is affected when water transport rates are decreased by inhibition of AQP3 function in Caco-2 cells incubated for 21 days. Even when AQP3 function was inhibited by 60% with HgCl_2_, there were no changes in warfarin permeability (Figure 2). AQPs allow water to move down an osmotic gradient [39,40]. However, even when mannitol was added to the basal side of Caco-2 cells to create an osmotic gradient facilitating apical-to-basal water movement, there were no changes in warfarin permeability (Figure 2B and Table 2). It has been reported that under normal conditions without shaking of Transwell plates, the permeability of Caco-2 cells to the lipophilic drug warfarin is decreased due to the thickness of the unstirred water layer [41]. It is also known that warfarin permeability can be increased by increasing the intensity of shaking to decrease the thickness of the unstirred water layer [42]. However, even with shaking, inhibition of AQP3 function had no effect on warfarin permeability (Figure 2B and Table 2). Furthermore, treatment with phloretin, whose site of action in inhibiting AQP3 function differs from that of HgCl_2_, did not affect warfarin permeability (Figure 2E and Table 2). Even the permeabilities of drugs with properties different from those of warfarin (i.e., antipyrine, atenolol, and furosemide) [31] were not affected by inhibition of AQP3 function (Figure 2D and Table 2). The above findings indicate that inhibition of AQP3 function has no effect on GI tract permeability to various drugs.

We next examined the effects of decreased cell membrane AQP3 expression on GI tract drug permeability. When the protein expression of AQP3 was decreased by 30% by PGE_2_ treatment, the P_app_ of warfarin was approximately twofold higher than the control level (Figure 3A,B). As observed for warfarin, the P_app_ of antipyrine was also increased by PGE_2_ treatment. In addition, tight junctions remained well-formed when PGE_2_ was added (Figure 3B). The above findings demonstrate that when AQP3 expression is decreased by 30% by PGE_2_ treatment, the rate of drug absorption in the GI tract increases.

As stated earlier, a PGE_2_-induced increase in drug permeability was observed for two types of drugs with different properties. It may therefore be concluded that the observed PGE_2_-induced increases in permeability were dependent not on the drug but rather on changes in the cell membrane. It has been reported that decreases in P-gp expression change the membrane conformation and increase membrane fluidity [33]. It has also been reported that drug permeability increases when membrane fluidity increases [34]. In fact, when P-gp expression was decreased by indomethacin, the permeability to warfarin and antipyrine increased (Figure 5). Given these findings, we analyzed fluorescence depolarization to examine whether the increases in membrane drug permeability due to decreased AQP3 expression were attributable to changes in cell membrane fluidity. We found that when AQP3 expression was decreased by 30% via PGE_2_ treatment, membrane fluidity increased by 15% compared with the control level (Figure 6A). Furthermore, when cell membrane fluidity was increased by boric acid/EDTA treatment [34,43] to approximately the same level as that achieved with PGE_2_ treatment, warfarin permeability increased to a level twofold higher than the control level; this increase was approximately the same magnitude as that observed with PGE_2_ treatment (Figure 6B). This evidence provides strong support for our hypothesis that increased drug permeability due to decreased AQP3 expression is attributable to increases in membrane fluidity rather than changes in water transport rates. The above results indicate that when AQP3 expression is decreased in the GI tract, the resulting increases in cell membrane fluidity lead to increases in permeability to drugs. Since AQP3 is expressed in various organs, further study is needed as to whether this result also occurs in other organs as well.

Conventionally, it has been thought that decreases in P-gp expression decrease transport capacity and thus lead to increased GI absorption of P-gp substrate drugs. However, the results of this study suggest that increased drug absorption due to decreased P-gp expression may involve increased drug permeability associated with increased membrane fluidity in addition to decreased excretory capacity. This may also hold true for non-P-gp substrate drugs, as our findings indicate that increases in membrane fluidity due to reductions in P-gp expression can lead to increased absorption rates.

It appears that there have been no studies on the relationship between the rate of water transport and the rate of drug absorption via passive transport. The results of this study reveal that water transport rates have little effect on drug absorption via passive transport. Our findings also indicate that although transmembrane proteins such as AQP3 do not themselves transport drugs, changes in their expression levels can alter membrane fluidity and thus affect drug absorption rates. AQP expression levels are affected by hormones [44,45], inflammatory cytokines [46,47], drugs [48,49], and other factors. The results of this study suggest that drug absorption can be accelerated in individuals being treated with drugs that decrease GI AQP3 expression or in those with pathological conditions characterized by decreased GI AQP3 expression, which we believe to be an important finding.

## 4. Materials and Methods

### 4.1. Cell Culture

Caco-2 cells (DS Pharma Biomedical Co., Ltd., Tokyo, Japan) were maintained in high-glucose Dulbecco’s modified Eagle’s medium (DMEM) supplemented with 10% fetal bovine serum, 100 U/mL penicillin G potassium, 100 μg/mL streptomycin, and 1% nonessential amino acids [50]. Cells were plated on a 24-well Transwell plate or 6-well Transwell plate at a density of 8 × 10^4^ cells/cm^2^ and incubated in a CO_2_ incubator at 37 °C for 21 days. Experiments were performed using cells that had previously been passaged 50 to 55 times. The integrity of the monolayer was measured by determining the TEER with an Epithelial Voltohmmeter (EVOM2, World Precision Instruments, Sarasota, FL, USA) [51].

### 4.2. Preparation of Samples for Western Blotting

Caco-2 cells were plated in 6-well Transwell plates and incubated for 3, 7, 14, and 21 days. The cells were lysed in lysis buffer (10 mM Tris, 150 mM NaCl, 8.5 μM leupeptin, 1 mM PMSF, 2.5 mM Na_3_VO_4_, and 0.5% NP-40; pH 7.2) and allowed to stand at 4 °C for 30 min. The lysates were then centrifuged at 15,000× *g* at 4 °C for 15 min and used as samples for western blotting [18].

### 4.3. Western Blotting

The samples were separated using SDS-PAGE, and the proteins were transferred to a polyvinylidene difluoride membrane. The membrane was blocked in blocking buffer and incubated with the following primary antibodies: Rabbit anti-rat AQP3 antibody (Alomone Labs, Jerusalem, Israel), C219 monoclonal antibody (Covance, Princeton, NJ, USA), and mouse anti-rabbit GAPDH antibody (Chemicon, Temeclula, CA, USA). The membrane was incubated with secondary antibodies, and the antibodies were detected with ECL Prime detection reagent (GE Healthcare, Milwaukee, CA, USA). The proteins were visualized using an LAS-3000 Mini Lumino image analyzer (Fujifilm, Tokyo, Japan).

### 4.4. Glycerol Permeability Assay

Caco-2 cells were plated in 24-well Transwell plates and incubated for 7, 14, and 21 days. After being removed from the culture medium, the cells were treated with HBSS (pH 6.0) on the apical side and HBSS (pH 7.4) on the basal side and preincubated at 37 °C in an atmosphere of 5% CO_2_ for 15 min. The cells were then treated on the apical side with glycerol (final concentration: 50 mM), a combination of glycerol and HgCl_2_ (final concentration: 10 µM), or a combination of glycerol and phloretin (final concentration: 100 µM). Samples were collected from the basal side up to 90 min after treatment, and the concentration of glycerol was determined using a glycerol colorimetric assay kit (Cayman Chemical, Ann Arbor, MI, USA) [52,53]. The P_app_ was then calculated based on the amount of glycerol that had permeated to the basal side [54].

### 4.5. Drug Permeability Assay

Caco-2 cells were plated in 24-well Transwell plates and incubated for 21 days. The cells were treated on the apical side with warfarin (final concentration: 100 µM), antipyrine (final concentration: 100 µM), atenolol (final concentration: 100 µM), or furosemide (final concentration: 100 µM), and samples were collected from the apical or basal side. The drug concentrations were then determined by HPLC to calculate the P_app_ values. The same assay was carried out on cells treated with HgCl_2_, phloretin, prostaglandin E_2_ (PGE_2_; final concentration: 10 µM), or indomethacin (final concentration: 0.4 µM). Also included in the assays were cells that were treated on the basal side with mannitol (final concentration: 100 mM) [55,56] and samples that were stirred on a plate mixer during the assay [57].

### 4.6. HPLC

The HPLC apparatus consisted of a Waters 2695 Separation Module (Waters, Tokyo, Japan) and a Waters 2489 UV/Visible Detector (Waters), and the measured data were recorded and analyzed using Empower analysis software (Waters). We used an Inertsil C18 ODS-3 column (mean particle size: 5 μm, 4.6 × 250 mm, GL Sciences Inc., Tokyo, Japan). The drug concentrations were calculated based on the absolute calibration curve method, and the response was found to be linear over the calibration range (from 4 µM to 100 µM) with a correlation coefficient of 0.999 (Table 3).

### 4.7. Lucifer Yellow Permeability Assay

Caco-2 cells were plated in 24-well Transwell plates and incubated for 21 days. The cells were then treated on the apical side with lucifer yellow (final concentration: 1000 µg/mL) or a combination of lucifer yellow and PGE_2_. Samples were collected from the basal side at 120 min after treatment. A fluorescence plate reader (Tecan GENios plate reader, Tecan, Salzburg, Austria) was used to measure the intensity of fluorescence at an emission wavelength of 535 nm and an excitation wavelength of 485 nm to calculate the permeability to lucifer yellow.

### 4.8. Measurement of Cell Membrane Fluidity

Caco-2 cells were plated in 6-well Transwell plates and incubated for 21 days. The cells were then treated on the apical side with boric acid (1%)/EDTA (0.05%), HgCl_2_, or PGE_2_. At 60 min after treatment, the cells were separated and suspended in PBS (2 × 10^5^ cells/mL). The cell suspension was then treated with 1,6-diphenyl-1,2,5-hexatriene (DPH)-tetrahydrofuran (final concentration: 1 µM) and allowed to stand at room temperature for 30 min. Fluorescence polarization was measured at an emission wavelength of 430 nm and an excitation wavelength of 360 nm using an Infinite 200PRO (Tecan). Membrane fluidity was calculated using the following formula:

r = () r = (I_//_ − I)/(I_//_ + 2I_⊥_)

where r is the fluorescence polarization, I_//_ is the fluorescence intensity parallel to the direction of the excitation beam, and I_⊥_ is the fluorescence intensity perpendicular to the direction of the excitation beam [58,59].

### 4.9. Statistical Analyses

The numerical data are expressed as the mean ± standard deviation (SD). Significance was examined using Student’s *t*-test or Dunnett’s test for multiple comparisons. Differences with a *p* < 0.05 were considered to be statistically significant.

## Figures and Tables

**Table 1:** P_app_ of glycerol during Caco-2 cell differentiation.

	Glycerol (10^−6^ cm/s)	Glycerol + HgCl_2_ (10^−6^ cm/s)
7 days	2380 ± 715	2172 ± 779
14 days	1986 ± 1222	929 ± 101 *
21 days	1000 ± 148	415 ± 66 **

Caco-2 cells were plated in Transwell plates and incubated for 7, 14, or 21 days. Glycerol alone or a combination of glycerol and HgCl_2_ was added to the apical side of the Transwell plate. The concentration of glycerol on the basal side was measured, and the P_app_ was then calculated. The data are presented as the mean ± SD for six experiments. Dunnett’s test: * *p* < 0.05 and ** *p* < 0.01 vs. the day 7 value.

**Table 2:** Effect of inhibition of AQP3 function on the P_app_ of warfarin.

Condition	Warfarin (10^−6^ cm/s)	Warfarin + HgCl_2_ (10^−6^ cm/s)
Normal condition	25.2 ± 4.7	24.2 ± 1.9
Osmotic pressure difference condition	24.1 ± 2.2	24.2 ± 4.4
Stirring condition	54.6 ± 1.8	53.3 ± 5.4

Caco-2 cells were plated in Transwell plates and incubated for 21 days. (1) Normal condition: Warfarin alone or a combination of warfarin and HgCl_2_ was added to the apical side of the Transwell plate. (2) Osmotic pressure difference condition: Warfarin alone or a combination of warfarin and HgCl_2_ was added to the apical side of the Transwell plate, and mannitol was added to the basal side. (3) Stirring condition: Warfarin alone or a combination of warfarin and HgCl_2_ was added to the apical side, and the Transwell plate was shaken on a plate mixer. The concentration of warfarin on the basal side was measured, and the P_app_ was then calculated. The data are presented as the mean ± SD for six experiments.

**Table 3:** HPLC conditions.

Drug	Mobile Phase	Wavelength (nm)	Flow Rate (mL/min)	Retention Time (min)
Warfarin	20 mM phosphate buffer:methanol = 55:45	308	1.0	10.0
Antipyrine	20 mM phosphate buffer:methanol = 70:30	254	1.0	12.9
Atenolol	50 mM phosphate buffer:acetonitrile = 90:10	226	1.0	5.2
Furosemide	50 mM phosphate buffer:methanol = 60:40	280	1.2	6.7

## Abbreviations

AQPs	Aquaporins
GI	Gastrointestinal
PGE_2_	Prostaglandin E_2_
P-gp	P-glycoprotein
CYP	Cytochrome P450
TEER	Transepithelial Electrical Resistance
P_app_	Apparent Permeability Coefficient
GAPDH	Glyceraldehyde-3-Phosphate Dehydrogenase
BCS	Biopharmaceutics Classification System
DMEM	Dulbecco’s Modified Eagle’s Medium
SD	Standard Deviation
